# Prediction of flare following remission and treatment withdrawal in early rheumatoid arthritis: post hoc analysis of a phase IIIb trial with abatacept

**DOI:** 10.1186/s13075-022-02735-8

**Published:** 2022-02-16

**Authors:** Harris A. Ahmad, Joshua F. Baker, Philip G. Conaghan, Paul Emery, Thomas W. J. Huizinga, Yedid Elbez, Subhashis Banerjee, Mikkel Østergaard

**Affiliations:** 1 Bristol Myers Squibb, Princeton, NJ USA; 2 Philadelphia Veteran’s Administration Medical Center and the University of Pennsylvania, Philadelphia, PA USA; 3 Leeds Institute of Rheumatic and Musculoskeletal Medicine, University of Leeds and NIHR Leeds Biomedical Research Centre, Leeds, UK; 4 Leiden University Medical Center, Leiden, Netherlands; 5 Deepscover, Puteaux, France; 6 Copenhagen Center for Arthritis Research, Center for Rheumatology and Spine Diseases, Rigshospitalet and Department of Clinical Medicine, University of Copenhagen, Copenhagen, Denmark

**Keywords:** Rheumatoid arthritis, Disease-modifying antirheumatic drugs (DMARDs), Magnetic resonance imaging (MRI), Abatacept, Flare

## Abstract

**Background:**

Drug-free remission is a desirable goal in rheumatoid arthritis (RA) for both patients and clinicians. The aim of this post hoc analysis was to investigate whether clinical and magnetic resonance imaging (MRI) variables in patients with early RA who achieved remission with methotrexate and/or abatacept at 12 months could predict disease flare following treatment withdrawal.

**Methods:**

In the AVERT study of abatacept in early RA, patients with low disease activity at month 12 entered a 12-month period with all treatment discontinued (withdrawal, WD). This post hoc analysis assessed predictors of disease flare at WD+6months (mo) and WD+12mo of patients with Disease Activity Score in 28 joints (DAS28)-defined remission (DAS28[C-reactive protein (CRP)] <2.6) at withdrawal using univariate and multivariable regression models. Predictors investigated included the Health Assessment Questionnaire–Disability Index (HAQ-DI), pain, Patient Global Assessment; MRI synovitis, erosion, bone edema, and combined (synovitis + bone edema) inflammation scores.

**Results:**

Remission was achieved by 172 patients; 100 (58%) and 113 (66%) patients had experienced a flare at WD+6mo and WD+12mo, respectively. In univariate analyses, higher HAQ-DI and MRI synovitis, erosion, bone edema, and combined inflammation scores at WD were identified as potential predictors of flare (*P* ≤ 0.01). In multivariable analysis, high scores at WD for HAQ-DI and MRI erosion were confirmed as independent predictors of flare at WD+6mo and WD+12mo (*P* < 0.01).

**Conclusion:**

In patients with early RA achieving clinical remission, patient function (HAQ-DI), and MRI measures of bone damage (erosion) predicted disease flare 6 and 12 months after treatment withdrawal. These variables may help identify patients with early RA in clinical remission as candidates for successful treatment withdrawal.

**Trial registration:**

ClinicalTrials.gov, NCT01142726 (date of registration: June 11, 2010)

**Supplementary Information:**

The online version contains supplementary material available at 10.1186/s13075-022-02735-8.

## Background

Rheumatoid arthritis (RA) is a debilitating inflammatory joint disease that results in structural joint damage and progressive disability if left untreated [1]. The burden of RA is considerable and includes pain, fatigue, reduced quality of life, and substantial socioeconomic costs [1, 2].

Early treatment of RA with disease-modifying antirheumatic drugs (DMARDs) is recommended to reduce inflammation, relieve symptoms, and halt or minimize structural progression that may lead to disability [3–5]. A treat-to-target approach [6] has been widely adopted by physicians with the aim of achieving remission or, if not possible, low disease activity through close monitoring, medication adjustment, and the use of biologic (b) DMARDs when indicated.

Drug-free remission is a highly desirable goal for both patients and physicians. Although the tapering or discontinuation of bDMARDs is often recommended in patients with sustained remission [4], complete withdrawal of RA therapy may be possible in some patients without inducing disease flares. Modern imaging techniques, soluble biomarkers, and physician/patient-reported measures offer the potential to predict such flares. Ultrasound has been identified as a possible clinically applicable predictive tool for flares but in relatively small, non-randomized studies following the tapering or discontinuation of bDMARDs [7–9]. Data for biomarkers as predictive tools are conflicting [10] and data for physician- and patient-reported measures are lacking.

Magnetic resonance imaging (MRI) has been used to assess the severity of joint damage and inflammation as well as response to treatment in clinical trials and real-world practice. Studies have shown correlations between reduction in MRI-assessed inflammation and reduced joint damage [11, 12] and have also demonstrated that MRI scores above (or below) a specific cut-off may be predictive of radiographic progression and/or low disease activity in patients with RA [13–16]. Identification of factors that predict flare could assist in determining which patients are suitable candidates for complete treatment withdrawal and aid individualized treatment decisions.

The T-cell costimulatory modulator, abatacept, approved for treatment of RA, halts the production of autoantibodies and proinflammatory cytokines by interrupting the cycle of T-cell activation initiated in RA. The *A*ssessing *V*ery *E*arly *R*heumatoid arthritis *T*reatment (AVERT) study of patients with early, active RA demonstrated the proportion of patients with Disease Activity Score in 28 joints (C-reactive protein) (DAS28[CRP])-defined remission (DAS28[CRP] <2.6) was significantly higher following 12 months of treatment with abatacept plus methotrexate (MTX) versus MTX alone [17]. Additionally, a significantly higher proportion of patients treated with abatacept plus MTX versus MTX alone maintained drug-free remission for 6 months after withdrawal of all RA treatment [17]. Furthermore, the majority of patients experienced a disease flare within 6 months of treatment withdrawal and few patients sustained major responses for 1 year [18].

The objective of this post hoc analysis of the AVERT study was to investigate whether specific patient and disease characteristics, including MRI findings, of patients in DAS28(CRP)-defined remission at 12 months could be used to predict disease flare following treatment withdrawal of abatacept plus MTX, abatacept monotherapy, or MTX alone. Predefined cut-offs in patient-reported outcome (PRO) and MRI scores based on earlier literature were evaluated as predictors of flare.

## Methods

### Study design and patient population

This was a post hoc analysis of the AVERT (NCT01142726, June 11, 2010) study [17]. AVERT was a phase IIIb, randomized, active-controlled 24-month study in adult patients with early (≤2 years), active RA consisting of a 12-month double-blind treatment period and a subsequent treatment withdrawal period (see Supplementary Fig. 1 in Additional File 1). All patients in AVERT satisfied the 2010 American College of Rheumatology/European League Against Rheumatism classification criteria for RA [17, 19] and were anti-cyclic citrullinated peptide (anti-CCP) positive. Details of sample size, power considerations, and methods for primary and secondary analyses in AVERT have previously been reported [17]. All patients who discontinued prior to completing the treatment or withdrawal period were imputed as non-responders for the month 12 or 18 analyses [17]. Patients enrolled in AVERT were MTX-naive or received MTX (≤10 mg/week) for ≤4 weeks with no MTX for 1 month prior to enrolment [17]. Patients were randomized to weekly subcutaneous abatacept 125 mg plus MTX (*n* = 119), abatacept 125 mg plus placebo (*n* = 116), or MTX plus placebo (*n* = 116) on day 1. MTX was initiated at 7.5 mg/week and titrated to 15–20 mg/week within 6–8 weeks [17].

For inclusion in the post hoc analysis, patients were required to have achieved DAS28(CRP)-defined remission [20] at month 12 and to have entered a subsequent 12-month withdrawal period in which all treatment was discontinued. Data from the three treatment arms in the double-blind period were pooled to increase the sample size for this analysis since it was believed that predictors of flare after treatment withdrawal should be independent of treatments used to achieve remission before drug discontinuation. This study was carried out in accordance with the Declaration of Helsinki. The AVERT study protocol was approved by the Institutional Review Board or Independent Ethics Committee at each site [17]. All study participants provided informed consent for involvement in the study.

### Study assessments

Patient and disease characteristics were assessed at withdrawal (WD; month 12), including CRP, DAS28(CRP), Health Assessment Questionnaire–Disability Index (HAQ-DI total score 0–3), pain (visual analog scale [VAS 0–100 mm scale]), Patient Global Assessment (VAS 0–100 mm scale), physical function (Short Form-36 subscale 0–100), Physician Global Assessment (VAS 0–100 mm scale), RA symptom duration, Swollen 28-Joint Count (SJC[28]), and Tender 28-Joint Count (TJC[28]). Age, weight, and RA symptom duration were taken from the AVERT study baseline. Furthermore, the following previously described MRI scores [13, 16] were available from WD: synovitis (total score 0–21), erosion (total score 0–230), bone edema (total score 0–69), unweighted combined inflammation (synovitis score + bone edema score), and weighted combined inflammation (synovitis score + 2x bone edema score). MRI scores were determined based on the Outcome Measures in Rheumatology RA MRI scoring system (OMERACT RAMRIS) [21] on MRI of the dominant wrist and metacarpophalangeal joints. As previously reported, the combined MRI inflammation score (synovitis + 2x bone edema) was weighted to account for the coefficient for bone edema being stronger than that of synovitis in multivariable models predicting X-ray and MRI progression [13].

Disease flare was assessed at WD+6months (mo; month 18) and WD+12mo (month 24) and was defined as a doubling of TJC(28) and SJC(28), increase in DAS28(CRP) ≥1.2 relative to the WD visit, or investigator’s judgment of RA flare [17].

### Assessment of association between WD clinical and MRI variables of interest and flare status at WD+6mo and WD+12mo

The association between demographic, clinical, and MRI variables at WD and subsequent flares at WD+6mo and WD+12mo was evaluated (see Supplementary Fig. 1 in Additional File 1). WD variables were analyzed as continuous measures and dichotomous variables of interest by using predefined cut-offs. A HAQ-DI cut-off score of >0.5 was used as an indicator of impaired physical function as a HAQ-DI score ≤0.5 has previously been considered an indicator of good physical function [22]. In line with Boolean criteria for remission, a cut-off score of >10 for patient pain and Patient Global Assessment scores (both VAS 0–100 mm scales) was used to indicate lack of remission status (based on study median values) [22]. Based on thresholds predictive of radiographic progression in previous studies, the following MRI cut-off scores were used: synovitis >3 [13], erosion >2 [16], bone edema >3 [13], unweighted combined inflammation >3 [16], and weighted combined inflammation >9 [13].

### Statistical analyses

WD patient demographic and disease characteristics stratified by flare status at WD+6mo and WD+12mo were described. Differences between patient and disease characteristics at WD in patients with and without flare at WD+6mo and WD+12mo were estimated using a Student’s *t*-test for equality of means (continuous variables) or a chi-square test (categorical variables). No correction for multiple testing was performed. Data from WD (or AVERT study baseline for age, weight, and duration of RA) were standardized to have a mean equal to zero and a standard deviation (SD) equal to one and were compared by the estimated differences between the flare versus no flare groups. *P* values of comparison were calculated by performing a Student’s *t* test. To assess the relationship of disease characteristics and MRI scores at WD with flare status at WD+6mo and WD+12mo, data from WD were standardized to have a mean equal to zero and an SD equal to one. Odds ratios (ORs) and *P* values were calculated from a logistic regression model for PROs of interest (HAQ-DI, patient pain, Patient Global Assessment), MRI measures, and DAS28(CRP) (to rule out any association with flare for patients in DAS28[CRP]-defined remission) by flare status. The scores at WD were the independent variables and flare at WD+6mo and WD+12mo were dependent variables. Statistical significance was set at *P* < 0.05.

Furthermore, the proportion of patients who experienced a flare or no flare at WD+6mo and WD+12mo, stratified by prespecified cut-off scores for PRO and MRI scores, was determined. Univariate logistic regression models were conducted for comparisons of flare rates above and below the predefined PRO and MRI cut-off scores to determine ORs with 95% confidence intervals (CIs) and associated *P* values. Finally, a multivariable logistic regression model, adjusted for treatment arm, was used to determine whether PRO and MRI scores at WD were independent predictors of flare at WD+6mo and WD+12mo.

## Results

Data were available for 172 patients with DAS28(CRP)-defined remission at WD across the three treatment arms (abatacept plus MTX, *n* = 70/119; abatacept monotherapy, *n* = 49/116; and MTX alone, *n* = 53/116). Overall, by WD+6mo, 100 patients (58%) had experienced a flare (abatacept plus MTX, *n* = 41/70; abatacept monotherapy, *n* = 28/49; and MTX alone, *n* = 31/53); by WD+12mo, this increased to 113 patients (66%) (abatacept plus MTX, *n* = 47/70; abatacept monotherapy, *n* = 30/49; and MTX alone, *n* = 36/53).

### Clinical and MRI variables at WD in patients who experienced a flare versus patients who did not experience a flare at WD+6mo and WD+12mo

WD patient demographic characteristics were generally well-balanced across patients stratified by flare status at WD+6mo and WD+12mo (Table 1). HAQ-DI scores were significantly higher at WD for patients who experienced a flare compared to patients who did not experience a flare both at WD+6mo and WD+12mo (*P* = 0.0088 and 0.0095, respectively). Statistically significant differences in all MRI measures were observed for patients who experienced a flare compared to patients who did not experience a flare at WD+6mo (*P* ≤ 0.01; Table 1) and most MRI measures at WD+12mo (*P* ≤ 0.01 for all measures, except synovitis where *P* = 0.0107; Table 1).

**Table 1:** Demographic and disease characteristics for all patients at WD and stratified by flare status

	Flare status at WD+6mo			Flare status at WD+12mo			Overall
Characteristic at WD	Flare (***n*** = 100)	No flare (***n*** = 72)	***P***	Flare (***n*** = 113)	No flare (***n*** = 59)	***P***	***N*** = 172
Age, years^a^	45.3 (12.7)	46.7 (11.9)	0.4757	45.4 (12.4)	46.8 (12.2)	0.4930	45.9 (12.3)
Weight, kg^a^	69.9 (14.3)	73.3 (15.9)	0.1403	70.0 (13.7)	73.9 (17.2)	0.1426	71.3 (15.1)
Female, *n* (%)^a^	78 (78.0)	53 (73.6)	0.5051	88 (77.9)	43 (72.9)	0.4655	131 (76.2)
White, *n* (%)^a^	89 (89.0)	55 (76.4)	0.0271	99 (87.6)	45 (76.3)	0.0558	144 (83.7)
RA symptom duration, years^a^	0.6 (0.6)	0.5 (0.4)	0.0463	0.6 (0.5)	0.5 (0.4)	0.3325	0.5 (0.5)
RF+, *n* (%)	88 (88.0)	59 (81.9)	0.6360	100 (88.5)	47 (79.7)	0.4063	166 (96.5)
ACPA+, *n* (%)	93 (93.0)	64 (88.9)	0.9511	105 (92.9)	52 (88.1)	0.9880	161 (93.6)
TJC (of 28 joints)	0.4 (0.8)	0.4 (0.6)	0.4475	0.4 (0.8)	0.4 (0.6)	0.4547	21.9 (14.6) *n* = 171
SJC (of 28 joints)	0.2 (0.7)	0.5 (1.1)	0.0305	0.3 (0.8)	0.4 (1.1)	0.3844	15.7 (12.0) *n* = 171
CRP, mg/mL	4.1 (5.7)	3.2 (4.1)	0.2442	3.9 (5.5)	3.3 (4.4)	0.4648	14.6 (19.8)
Physician Global Assessment, VAS 0–100 mm	4.5 (7.0)	5.0 (7.4)	0.6912	4.8 (7.2)	4.5 (7.1)	0.7858	57.0 (18.9) *n* = 170
HAQ-DI score (total score 0–3)	0.5 (0.5) *n* = 94	0.3 (0.4) *n* = 65	0.0088	0.4 (0.5) *n* = 107	0.2 (0.4) *n* = 52	0.0095	1.3 (0.7) *n* = 161
Pain, VAS 0–100 mm	14.6 (14.4) *n* = 94	13.2 (15.4) *n* = 66	0.5422	14.1 (14.0) *n* = 107	13.9 (16.5) *n* = 53	0.9340	58.3 (20.0) *n* = 161
Patient Global Assessment, VAS 0–100 mm	12.1 (14.7)	11.0 (13.5)	0.6092	11.6 (14.3)	11.7 (14.2)	0.9681	57.3 (21.5) *n* = 167
Physical function, SF-36	77.8 (19.7)	80.7 (23.4)	0.3733	78.0 (19.9)	80.9 (24.0)	0.3879	42.8 (25.7) *n* = 161
DAS28(CRP)	1.8 (0.4)	1.8 (0.5)	0.8519	1.8 (0.4)	1.8 (0.5)	0.8144	1.8 (0.4)
Treatment group, *n* (%)							
Abatacept + MTX	41 (41.0)	29 (40.3)	0.9928	47 (41.6)	23 (39.0)	0.9270	70 (40.7)
Abatacept	28 (28.0)	21 (29.2)	0.8905	30 (26.5)	19 (32.2)	0.4793	49 (28.5)
MTX	31 (31.0)	22 (30.6)	NR	36 (31.9)	17 (28.8)	NR	53 (30.8)
MRI synovitis score (total score 0–21)	3.7 (2.7) *n* = 92	2.7 (2.1) *n* = 63	0.0070	3.7 (2.6) *n* = 103	2.6 (2.1) *n* = 52	0.0107	NR
MRI erosion score (total score 0–230)	7.6 (6.1) *n* = 92	4.4 (3.7) *n* = 63	<0.0001	7.4 (6.0) *n* = 103	4.3 (3.6) *n* = 52	0.0001	NR
MRI bone edema score (total score 0–69)	2.1 (3.9) *n* = 92	0.6 (1.1) *n* = 63	0.0007	2.0 (3.8) *n* = 103	0.6 (1.0) *n* = 52	0.0004	NR
MRI unweighted combined inflammation score^b^	5.8 (5.8) *n* = 92	3.3 (2.7) *n* = 63	0.0003	5.6 (5.6) *n* = 103	3.1 (2.6) *n* = 52	0.0002	NR
MRI weighted combined inflammation score^c^	8.0 (9.5) *n* = 92	3.9 (3.5) *n* = 63	0.0003	7.6 (9.1) *n* = 103	3.7 (3.3) *n* = 52	0.0002	NR

Values are mean (standard deviation) unless indicated otherwise. *P* value for continuous variables is from a Student’s *t* test for equality of means. *P* value for categorical variables is from a chi-square test

^a^Indicates data was collected at Assessing Very Early Rheumatoid arthritis Treatment (AVERT) study baseline (not WD)

^b^Synovitis score + edema score

^c^Synovitis score + 2x edema score

*ACPA* anti-citrullinated protein antibody, *CRP* C-reactive protein, *DAS28* Disease Activity Score in 28 joints, *HAQ-DI* Health Assessment Questionnaire–Disability Index, *mo* months, *MRI* magnetic resonance imaging, *MTX* methotrexate, *NR* not relevant, *RA* rheumatoid arthritis, *RF* rheumatoid factor, *SJC(28)* Swollen 28-Joint Count, *SF-36* Short Form-36 subscale (0–100 scale), *TJC(28)* Tender 28-Joint Count, *VAS* visual analog scale, *WD* withdrawal

**Fig. 1:**
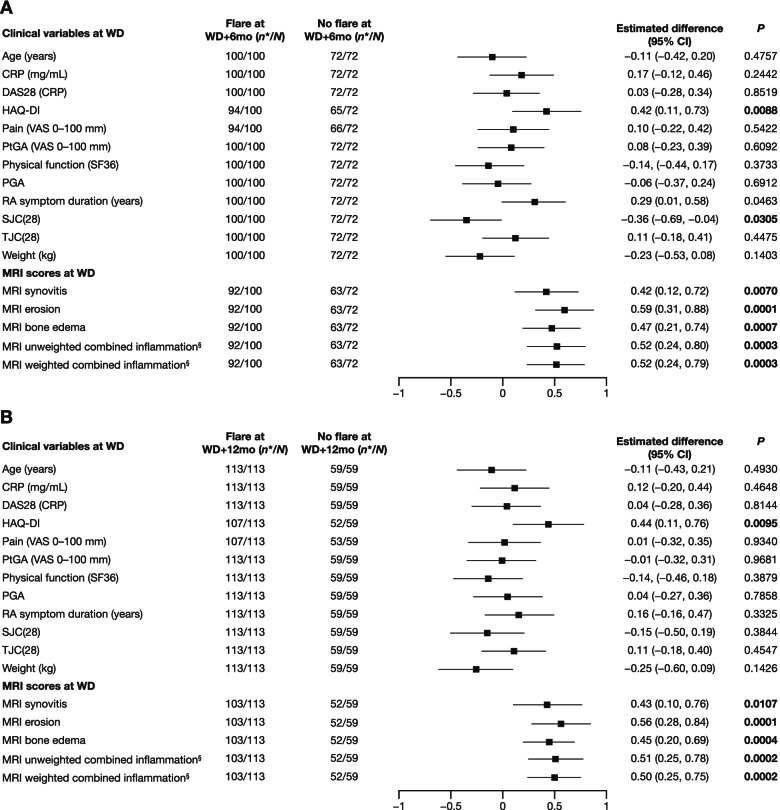
Standardized estimated difference between patients with flare versus no flare for clinical and MRI variables. Data are shown for patients with and without flare at **A** WD+6months (mo) and **B** WD+12mo. Data from WD (or at baseline for age, weight, and duration of rheumatoid arthritis [RA]) were standardized to have a mean equal to zero and a standard deviation (SD) equal to one and were compared by the estimated differences between flare versus no flare groups. Vertical line indicates limit of effect: positive data indicate effect, negative data or data that cross 0 indicate absence of effect. *P* values of comparison were calculated from Student’s *t* test; values in bold indicate statistical significance. *Number of subjects with WD data available. ^†^Higher SF-36 scores denote improvement in quality of life. ^‡^Synovitis score + edema score. ^§^Synovitis score + 2x edema score. *CI* confidence interval, *CRP* C-reactive protein, *DAS28* Disease Activity Score in 28 joints, *HAQ-DI* Health Assessment Questionnaire–Disability Index, *OR* odds ratio, *PGA* Physician Global Assessment, *PRO* patient-reported outcomes, *PtGA* Patient Global Assessment, *SF36* Short Form-36, *SJC(28)* Swollen 28-Joint Count, *TJC(28)* Tender 28-Joint Count, *VAS* visual analog scale, *WD* withdrawal

For patients who had experienced a flare by WD+6mo or WD+12mo (compared to no flare), the standardized estimated differences in HAQ-DI score and all MRI measures at WD were statistically significant (*P* < 0.02 for all; Fig. 1).

**Fig. 2:**
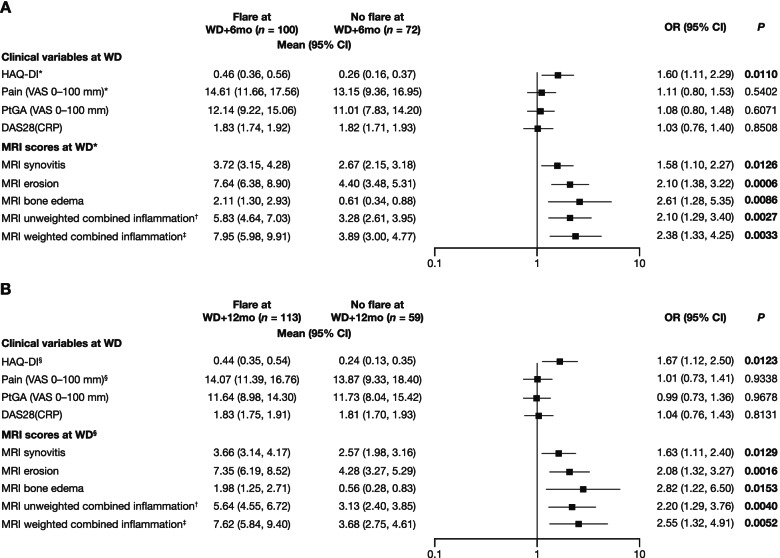
Clinical and MRI variables at WD and associated ORs for flare versus no flare. Data are shown for flare versus no flare at **A** WD+6mo and **B** WD+12mo. Data from WD were standardized (mean equal to zero and SD equal to one). Vertical line indicates limit of effect: positive data indicate effect, negative data or data that cross 1 indicate absence of effect. ORs (per one unit) and *P* values are from a univariate logistic regression model with scores at WD as the independent variables and flare at WD+6mo and WD+12mo as the dependent variable; bold *P* values indicate statistical significance. *HAQ-DI: *n* = 94 for flare at WD+6mo and *n* = 65 at WD+12mo; pain: *n* = 94 for flare at WD+6mo and *n* = 66 at WD+12mo; MRI: *n* = 92 for flare at WD+6mo and *n* = 63 at WD+12mo. ^†^Synovitis score + edema score. ^‡^Synovitis score + 2x edema score. ^§^HAQ-DI: *n* = 107 for flare at WD+6mo and *n* = 52 at WD+12mo; pain: *n* = 107 for flare at WD+6mo and *n* = 52 at WD+12mo; MRI: *n* = 103 for flare at WD+6mo and *n* = 52 at WD+12mo. *OR* odds ratio. See Fig. 1 for other definitions

The association of DAS28(CRP), PROs of interest, and MRI measures at treatment withdrawal with flare at WD+6mo and WD+12mo was examined by univariate logistic regression. A higher HAQ-DI score at WD was significantly associated with flare at WD+6mo (OR, per one unit, 1.60 [1.11, 2.29], *P* = 0.0110; Fig. 2A) and WD+12mo (OR 1.67 [1.12, 2.50], *P* = 0.0123; Fig. 2B). In contrast, DAS28(CRP), pain, and Patient Global Assessment scores at WD were not associated with flare at WD+6mo or WD+12mo. Higher MRI weighted combined inflammation scores at WD were significantly associated with flare at WD+6mo (OR 2.38 [1.33, 4.25], *P* = 0.0033; Fig. 2A) and WD+12mo (OR 2.55 [1.32, 4.91], *P* = 0.0052; Fig. 2B). All other MRI scores at WD in Fig. 2 were also significantly associated with flare at WD+6mo and WD+12mo.

### Dichotomized clinical and MRI variables at WD and their relationship with subsequent flare versus no flare at WD+6mo and WD+12mo

Patients were dichotomized according to predefined cut-off scores of PRO and MRI variables at WD. The association with flare at WD+6mo and WD+12mo based on these stratifications is shown in Supplementary Fig. 2 in Additional File 1. At both WD+6mo and WD+12mo, a higher proportion of patients with a HAQ-DI score of >0.5 experienced a flare compared with those who had a score of ≤0.5 (81% versus 51% and 88% versus 60%, respectively (Supplementary Fig. 2A in Additional File 1)). The difference in the proportion of patients experiencing a flare with pain scores above (versus below) the predefined cut-off score (10), was less pronounced (WD+6mo: 64% versus 55%; WD+12mo: 72% versus 63%), and this was also true for the Patient Global Assessment scores.

For all MRI measures, higher proportions of patients with scores above the predefined cut-offs experienced a flare than patients with scores below the predefined cut-offs (Supplementary Fig. 2B in Additional File 1). For example, 86% (*n*/*N* = 25/29) of patients who had MRI weighted combined inflammation scores above the predefined cut-off of 9 experienced a flare at WD+6mo, while 53% (*n*/*N* = 67/126) with scores below the cut-off experienced a flare.

Furthermore, univariate logistic regression analysis was used to assess the relationship between dichotomized PRO and MRI variables at WD and flare status at WD+6mo and WD+12mo. Above-cut-off scores for HAQ-DI and most MRI variables at WD were significantly associated with flare at WD+6mo and/or WD+12mo (Supplementary Fig. 3 in Additional File 1). HAQ-DI was the only PRO to show association with flare (WD+6mo: OR 4.04 [1.72, 9.46], *P* = 0.0013; WD+12mo: OR 4.97 [1.82, 13.57], *P* = 0.0018). Compared with the other measures evaluated, an MRI weighted combined inflammation score >9 showed the greatest odds of flare at both WD+6mo and WD+12mo (OR 5.50 [1.81, 16.72], *P* = 0.0027 and OR 5.52 [1.58, 19.20], *P* = 0.0073, respectively). Above cut-off scores for pain, Patient Global Assessment, MRI synovitis, and MRI unweighted combined inflammation scores were not significantly associated with flare at WD+6mo or WD+12mo.

**Fig. 3:**
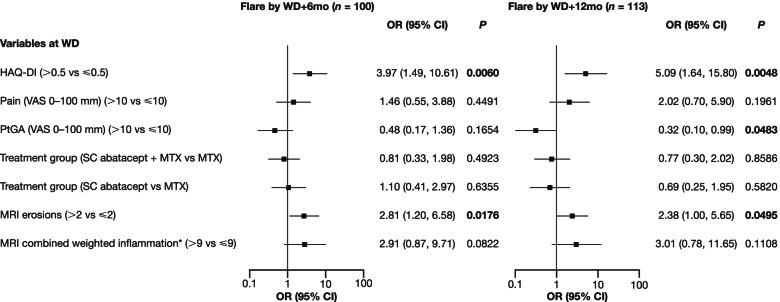
Multivariable logistic regression analysis assessing the value of cut-off scores for predicting flare. Analysis was performed for flare status at WD+6mo and WD+12mo. A multivariable logistic regression model, adjusted for treatment arm, determined whether patient-reported outcome (PRO) and MRI measures at WD were independent predictors of flare at WD+6mo and WD+12mo. *P* values in bold type indicate statistical significance. Vertical line indicates limit of effect: positive data indicate effect, negative data or data that cross 1 indicate absence of effect. *Synovitis score + (2x bone edema score). *MTX* methotrexate, *SC* subcutaneous. See Fig. 1 for other definitions

Finally, a multivariable logistic regression model, adjusted for the treatment arm, was applied to test independent WD predictors of subsequent flare. In this analysis, a HAQ-DI score >0.5 was shown to be independently associated with flare at both WD+6mo and WD+12mo (OR 3.97 [1.49, 10.61], *P* = 0.0060 and OR 5.09 [1.64, 15.80], *P* = 0.0048, respectively; Fig. 3). Similarly, an MRI erosion score >2 was shown to be independently associated with flare at both WD+6mo and WD+12mo (OR 2.81 [1.20, 6.58], *P* = 0.0176 and OR 2.38 [1.00, 5.65], *P* = 0.0495, respectively; Fig. 3). A weighted combined inflammation score >9 showed a trend towards being independently associated with flare at both WD+6mo and WD+12mo (OR 2.91 [0.87, 9.71], *P* = 0.0822 and OR 3.01 [0.78, 11.65], *P* = 0.1108, respectively; Fig. 3). Patient Global Assessment scores were only independently associated with flare at WD+12mo (OR 0.32 [0.10, 0.99], *P* = 0.0483), while pain was not independently associated with flare at either timepoint.

## Discussion

In this post hoc analysis of the AVERT study in patients with early, active RA, we identified predictors of disease flare in patients who discontinued all RA treatment after achieving DAS28(CRP)-defined remission at month 12. In multivariable analysis, HAQ-DI (physical function) and MRI-detected erosion (bone damage) scores at WD were found to be independent predictors of disease flare at WD+6mo and WD+12mo. MRI-detected weighted combined inflammation (incorporating synovitis and 2x bone edema) showed a trend towards independently predicting disease flare at WD+6mo and WD+12mo. These observations suggest that these measures may help guide physicians to make decisions with regard to drug withdrawal after remission is achieved in patients with RA treated with abatacept.

In RA, treatment withdrawal following the achievement of remission without subsequent disease flare (i.e., sustained drug-free remission) is a highly desirable goal [23]. Tools that could be incorporated into routine clinical practice to help characterize patients for whom sustained remission is more likely, or who are at a higher risk of flare, may help to guide treatment decisions. Recent studies have highlighted potential predictors of flare following treatment tapering or discontinuation in patients with long-standing RA and sustained remission, with several studies focusing on the utility of power Doppler ultrasound (PDUS) and MRI measures [7, 8, 13, 16].

Patients in the AVERT study had early disease and all RA treatment (including MTX and corticosteroids) was withdrawn in those with a low disease activity score after 12 months. The AVERT study demonstrated that a significantly higher proportion of patients treated with abatacept plus MTX, versus MTX alone, maintained drug-free remission for 6 months after the withdrawal of all RA treatment [17]. Additionally, baseline corticosteroid use and Patient Global Assessment scores were found to be predictive of a shorter time to RA flare after treatment withdrawal and for the achievement of DAS28(CRP)-defined remission after 6 months of retreatment with abatacept plus MTX, respectively [18]. Despite seropositivity being linked to predicting better efficacy of abatacept in the AMPLE (*A*batacept versus adali*M*umab com*P*arison in bio*L*ogic-naiv*E* rheumatoid arthritis subjects with background MTX) study [24], in the AVERT study, there was no link between withdrawal of abatacept and increased risk of flare in patients with anti-CCP positive RA [18]. In contrast to the previous AVERT study analysis, which explored whether clinical characteristics were associated with time to disease flare or with regaining disease control after treatment [18], the present analysis assessed which clinical characteristics were associated with flare after treatment withdrawal. We found several clinical characteristics to be associated with flare in univariate analyses: HAQ-DI, and MRI synovitis, erosion, bone edema, and weighted and unweighted combined inflammation scores. A previous analysis from the GO-BEFORE trial of bDMARD-naive patients with RA treated with tumor necrosis factor inhibitor (TNFi) therapy and/or MTX found that MRI synovitis, bone edema, and erosion independently correlated with physical function, pain, and Patient Global Assessment scores [25]. The current analysis found that both increased HAQ-DI scores (impaired physical function) and higher levels of MRI findings (inflammation or structural damage) were independently predictive of disease flare after treatment withdrawal. However, a previous study of RA treatment discontinuation after the achievement of remission in patients with recent-onset RA receiving conventional synthetic DMARDs identified low baseline HAQ-DI scores as a predictor for restarting treatment [26].

Studies have also shown the utility of synovitis scoring measured by PDUS for predicting the failure of bDMARD tapering and the identification of suitable patients for treatment tapering or discontinuation after the achievement of sustained remission with TNFi therapy [7, 8]. As in the present study, no association between demographic variables and subsequent disease relapse was found [8]. However, another study found no association between PDUS and flare following TNFi discontinuation [27]; the latter study reported that TNFi treatment initiation early in the disease course was the main predictor of successful discontinuation [27].

The ability of MRI to detect subclinical joint inflammation [13, 28–32] may explain our observation that MRI, but not laboratory measures of disease activity such as CRP or clinical measures such as SJC(28) or TJC(28), predicted risk of flare. As more data on predictors of flare after treatment taper or withdrawal are collected, a combination of clinical and imaging factors may be defined for the accurate identification of patients suitable for treatment withdrawal or those who would be at risk of flare. The costs of performing an MRI scan for this purpose would need to be balanced against potential savings in bDMARD usage [33] and the potential to spare patients unnecessary treatment.

The second stage of the present analysis was to test previously defined cut-off scores for their value in predicting flare. The cut-off scores for HAQ-DI (>0.5), pain, and Patient Global Assessment (both >10) tested in the current analysis were chosen based on prior evidence demonstrating these to be indicators of good physical function and Boolean remission [22]. Cut-off scores to test for MRI measures were chosen from two separate analyses. Baker et al. previously defined and validated thresholds of MRI synovitis and bone edema associated with low risk of radiographic progression in a subanalysis of data from randomized clinical trials of the TNFi golimumab in patients with RA (GO-BEFORE and GO-FORWARD studies) [13]; a cut-off score of ≤3 for MRI synovitis and bone edema was shown to identify patients at low risk of progression. In addition, a cut-off score of ≤9 for an MRI weighted combined inflammation score (synovitis score + 2x edema score) also identified patients with a very low risk of radiographic progression [13]. The cut-off scores for MRI synovitis and unweighted combined inflammation were developed by Brahe and colleagues during a dose-tapering study of patients with RA being treated with bDMARDs (the Danish A Dose OPTimization of biological therapy [ADOPT] study) [16]. As part of that analysis, receiver operator characteristic curves were generated to identify cut-off values for baseline variables. The exploratory analysis showed that a cut-off score of ≤2 for MRI erosions and ≤3 for MRI combined inflammation could be used to predict successful tapering of therapy for patients in sustained remission [16]. It should be noted that some of the MRI cut-off scores described above were based on predicting radiographic progression, whereas in the current analysis the cut-off scores were used to predict disease flares. The identification of important thresholds below which the safe withdrawal of effective treatments may be achieved is an important step forward in the precision use of therapies for RA.

Univariate analyses in the present study showed HAQ-DI and MRI synovitis, bone edema, erosion, and weighted and unweighted combined inflammation scores to be significantly predictive of flare 6 and 12 months following treatment withdrawal. Following multivariable analysis, we found HAQ-DI and MRI erosion scores to be predictors of disease flare at both 6 and 12 months following treatment withdrawal, while weighted combined inflammation showed a trend towards independently predicting disease flare. The finding that bone erosion was a predictor of disease flare in addition to inflammatory measures may indicate that those with RA-specific damage are also at higher risk of disease flare, perhaps related to a more severe disease phenotype. A recent post hoc analysis of the 2-year Danish IMAGINE-RA clinical trial (*n* = 171) showed baseline MRI osteitis (bone edema) and tenosynovitis to be independent predictors of 2-year MRI damage progression in patients with RA in clinical remission [34]. This further highlights the potential of MRI measures to guide an individualized approach to the management of RA.

Potential limitations of this study include the post hoc nature of the evaluation. The patient sample represented only a subgroup of the whole study population of AVERT and numbers were relatively small; thus, data should be interpreted with caution, as it may not be generalizable to other patient subgroups, different treatments, or the general RA population. Additionally, all patient data across the three treatment arms were pooled rather than stratified by treatment to provide a larger data set. As this study was conducted in patients with early RA, future studies will be needed to confirm whether the cut-off scores tested here would also predict disease flare in other RA populations or following the withdrawal or tapering of bDMARDs other than abatacept. There are several different definitions of flare (or relapse) in RA and, as such, results may vary slightly depending on which definition is used (and consequently which patients were included) [35–37].

Despite limitations, the current post hoc analysis had the strength of using data from a 2-year clinical trial comprising a 12-month treatment period followed by 12-month withdrawal period, in which patients were closely and systematically monitored. Furthermore, in AVERT, the withdrawal of all RA therapy (abatacept, background MTX, and glucocorticoids) allowed for the study of true drug-free remission. Finally, the testing of cut-offs for HAQ-DI, pain, Patient Global Assessment, and MRI measures provides a sense of how these measures may be used clinically to guide decisions surrounding WD or tapering of therapy in RA.

## Conclusions

In summary, physical function (HAQ-DI) and objective MRI measures of inflammation and damage (erosion) at treatment withdrawal were independent predictors of flare 6 and 12 months after cessation of treatment with abatacept in patients with early RA in DAS28(CRP)-defined remission. Cut-off scores of these variables were independent predictors of flare and may have the potential to help guide clinical decision making about continuing or withdrawing therapy in patients with RA in remission.

## Supplementary Information

**Additional file 1: Supplementary Fig. 1** *A*ssessing *V*ery *E*arly *R*heumatoid arthritis *T*reatment (AVERT) study and post hoc analysis design. **Supplementary Fig. 2** WD+6mo and WD+12mo flare rates for patients with PRO/MRI variables above/below predefined cut-off scores. **Supplementary Fig. 3** Univariate logistic regression analysis assessing relationship between cut-off scores at WD and flare status.

## Data Availability

Bristol Myers Squibb policy on data sharing may be found at https://www.bms.com/researchers-and-partners/independent-research/data-sharing-request-process.html

## Abbreviations

b: Biologic
CI: Confidence interval
CRP: C-reactive protein
DAS28: Disease Activity Score in 28 joints
DMARD: Disease-modifying antirheumatic drug
HAQ-DI: Health Assessment Questionnaire–Disability Index
mo: Months
MRI: Magnetic resonance imaging
MTX: Methotrexate
OMERACT: Outcome Measures in Rheumatology
OR: Odds ratio
PDUS: Power Doppler ultrasound
PRO: Patient-reported outcome
RA: Rheumatoid arthritis
RAMRIS: Rheumatoid Arthritis Magnetic Resonance Imaging scoring system
SD: Standard deviation
SJC28: Swollen 28-Joint Count
TJC28: Tender 28-Joint Count
TNFi: Tumor necrosis factor inhibitor
VAS: Visual analog scale
WD: Withdrawal
