# Supplementary material for: Prediction of flare following remission and treatment withdrawal in early rheumatoid arthritis: post hoc analysis of a phase IIIb trial with abatacept
Source: Arthritis Res Ther. 2022 Feb 16;24:47. doi: 10.1186/s13075-022-02735-8 (PMC8848810; doi:10.1186/s13075-022-02735-8)
Supplement: Supplementary file 1 — Additional file 1: Supplementary Fig. 1 Assessing Very Early Rheumatoid arthritis Treatment (AVERT) study and post hoc analysis design. Supplementary Fig. 2 WD+6mo and WD+12mo flare rates for patients with PRO/MRI variables above/below predefined cut-off scores. Supplementary Fig. 3 Univariate logistic regression analysis assessing relationship between cut-off scores at WD and flare status. [file 13075_2022_2735_MOESM1_ESM.docx]

# SUPPLEMENTARY INFORMATION


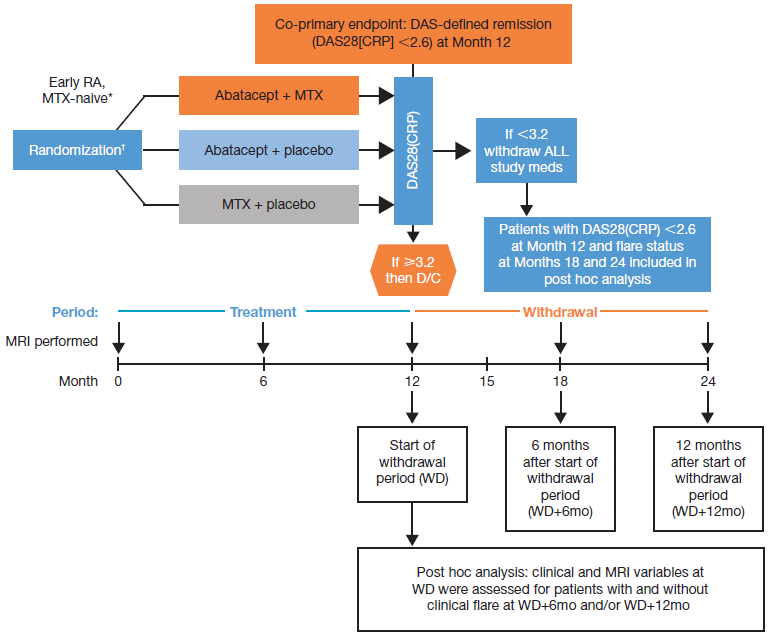


**Supplementary Figure 1.** Assessing Very Early Rheumatoid arthritis Treatment (AVERT) study and post hoc analysis design. Adapted from Emery P, et al. *Ann Rheum Dis* 2015;74:19–26 (published Open Access [CC BY-NC 4.0 license]).[17] *Prior methotrexate (MTX) use discontinued 4 weeks prior to enrollment. ^†^Randomization stratified by corticosteroid use at baseline.

*CRP* C-reactive protein, *DAS28* Disease Activity Score in 28 joints, *D/C* discontinued the study, *meds* medications, *mo* months, *MRI* magnetic resonance imaging, *RA* rheumatoid arthritis, *WD* withdrawal.


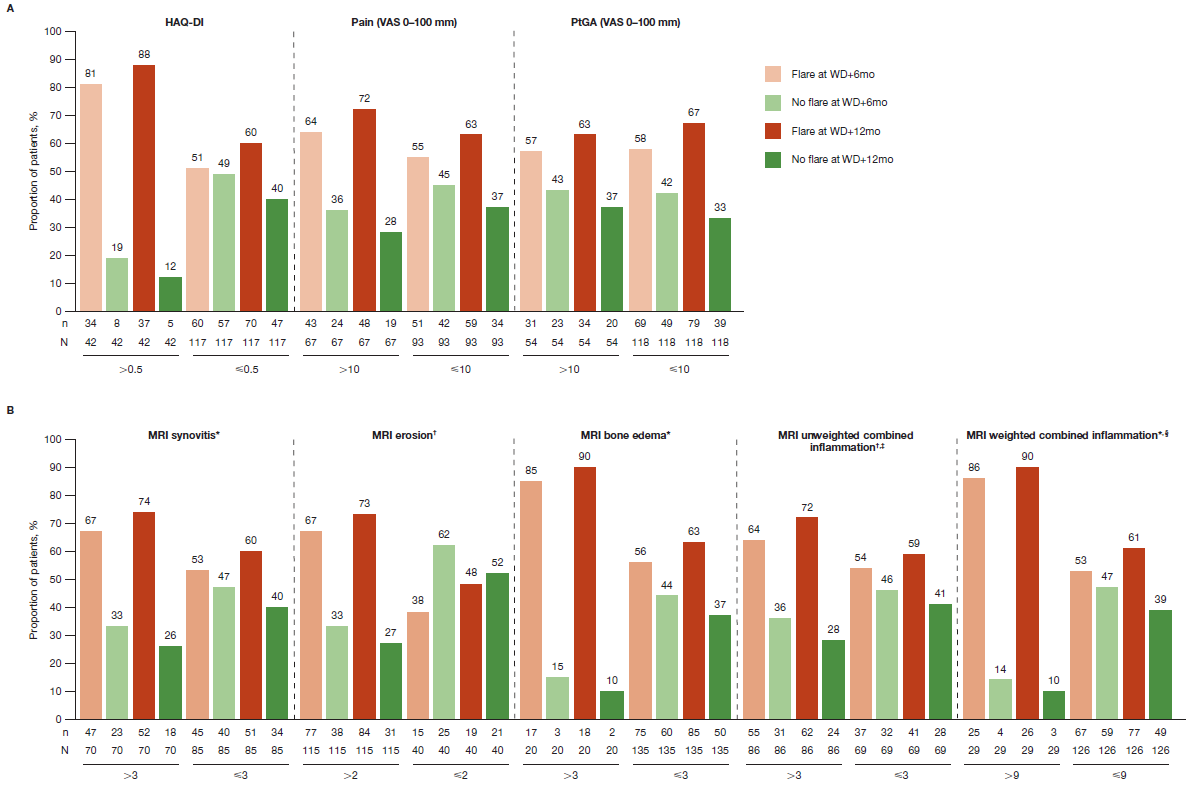


**Supplementary Figure 2.** WD+6mo and WD+12mo flare rates for patients with PRO/MRI variables above/below predefined cut-off scores. Panel (**A**) shows those with PROs and panel (**B**) shows those with MRI variables above/below predefined cut-off scores at WD. Patients were stratified by prespecified cut-off scores for PRO and MRI scores.

*Baker cut-off [13].

^†^Brahe cut-off [16].

^‡^Synovitis score + edema score.

^§^Synovitis score + 2x edema score.

*HAQ-DI* Health Assessment Questionnaire–Disability Index, *MRI* magnetic resonance imaging, *PRO* patient-reported outcomes, *PtGA* Patient Global Assessment, *VAS* visual analog scale, *WD* withdrawal.


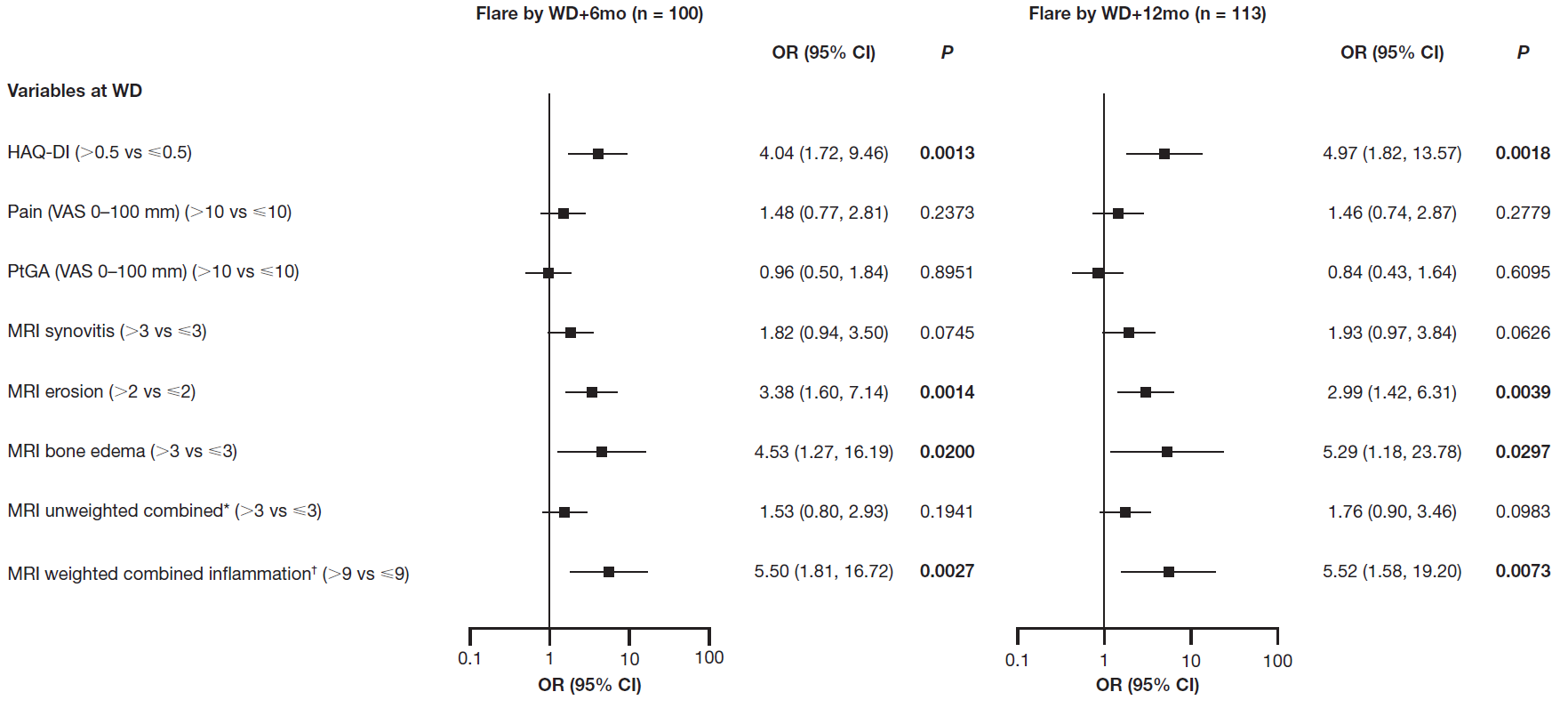


**Supplementary Figure 3.** Univariate logistic regression analysis assessing relationship between cut-off scores at WD and flare status. Flare status was assessed at WD+6mo and WD+12mo. Univariate logistic regression models were conducted to compare flare rates above and below the predefined PRO and MRI cut-off scores. Vertical line indicates limit of effect: positive data indicate effect, negative data or data that cross 1 indicate absence of effect. *P* values in bold type indicate statistical significance.

*Synovitis score + edema score.

^†^Synovitis score + 2x edema score.

*CI* confidence interval, *HAQ-DI* Health Assessment Questionnaire–Disability Index, *MRI* magnetic resonance imaging, *OR* odds ratio, *PRO* patient-reported outcomes, *PtGA* Patient Global Assessment, *VAS* visual analog scale, *WD* withdrawal.
